# Reframing Adult ADHD: A Salience-Arousal Regulation Phenotype

**DOI:** 10.1192/bjo.2026.11122

**Published:** 2026-06-30

**Authors:** Malcolm Scobbie

**Affiliations:** Harrow Health C.I.C, London, United Kingdom

## Abstract

**Aims:**

Adult ADHD remains largely defined by behavioural criteria developed for childhood presentations. In adult psychiatric practice, however, many individuals present with burnout, anxiety, or emotional exhaustion rather than overt attentional complaints. These patients often describe functioning well in some contexts but struggling disproportionately with routine, low-urgency, or poorly defined tasks. This work aims to explore whether adult ADHD is better conceptualised as a disorder of salience and arousal regulation rather than a simple deficit of attention or impulse control, and whether such a formulation better accounts for late diagnosis and masked presentations.

**Methods:**

This work draws on repeated clinical observations from general adult psychiatricpractice, including experience in adult ADHD assessment services, synthesised into anonymised composite vignettes to identify recurring patterns of cognition, behaviour, and emotional experience. These observations were considered alongside established executive, motivational, and salience-based models of ADHD, and contemporary neurobiological accounts of dopamine and noradrenaline modulation, sensory gating, and time perception, to develop a clinically coherent formulation. An exploratory evolutionary perspective was used cautiously to illustrate environmental mismatch rather than ancestral advantage.

**Results:**

Consistent features extended beyond standard diagnostic criteria and included reliance on urgency or interest to initiate tasks, development of highly personalised systems of working, rapid associative thinking alongside episodes of task paralysis, conversational working-memory bottlenecks, and difficulties maintaining awareness of objects or people once out of view. Many individuals also described strong internal value systems and high personal standards, with particular sensitivity to inefficiency or perceived unfairness. Hyperfocus and distractibility could be understood within a single salience-arousal framework rather than as opposing phenomena. Functional decline commonly occurred at points of developmental or occupational transition and following additional stressors, consistent with cumulative compensatory fatigue rather than late onset.

**Conclusion:**

Conceptualising adult ADHD as a regulation phenotype provides a clinically useful account of context-dependent functioning, burnout, and late diagnosis. This framework supports improved recognition of masked ADHD across adult services and encourages formulations that integrate pharmacological treatment with psychoeducation and environmental adaptation. Moving beyond checklist-driven assessment towards pattern-based clinical understanding may reduce secondary morbidity and improve long-term functional outcomes in adults with ADHD.

